# Metabotropic glutamate receptor 5 (mGluR5) regulates bladder nociception

**DOI:** 10.1186/1744-8069-8-20

**Published:** 2012-03-26

**Authors:** Lara W Crock, Kristina M Stemler, David G Song, Philip Abbosh, Sherri K Vogt, Chang-Shen Qiu, H Henry Lai, Indira U Mysorekar, Robert W Gereau IV

**Affiliations:** 1 Neuroscience Program, Washington University School of Medicine, St. Louis, MO, USA; 2 Medical Scientist Training Program, Washington University School of Medicine, St. Louis, MO, USA; 3 Department of Anesthesiology, Washington University Pain Center, St. Louis, MO, USA; 4 Department of Obstetrics and Gynecology, Washington University School of Medicine, St. Louis, MO, USA; 5 Saint Louis University School of Medicine, St. Louis, MO, USA; 6 Division of Urologic Surgery, Department of Surgery, St. Louis Veterans Affairs Medical Center, Washington University School of Medicine, St. Louis, MO, USA; 7 Department of Anesthesiology, Washington University School of Medicine, Campus Box 8054, St Louis, MO 63110, USA

**Keywords:** Nociception, Bladder, Visceromotor Response, Urinary Tract Infection, Metabotropic Glutamate Receptor

## Abstract

**Background:**

Interstitial cystitis/painful bladder syndrome (IC/PBS), is a severely debilitating chronic condition that is frequently unresponsive to conventional pain medications. The etiology is unknown, however evidence suggests that nervous system sensitization contributes to enhanced pain in IC/PBS. In particular, central nervous system plasticity of glutamatergic signaling involving NMDA and metabotropic glutamate receptors (mGluRs) has been implicated in a variety of chronic pain conditions. Here, we test the hypothesis that mGluR5 mediates both non-inflammatory and inflammatory bladder pain or nociception in a mouse model by monitoring the visceromotor response (VMR) during graded bladder distention.

**Results:**

Using a combination of genetic and pharmacologic approaches, we provide evidence indicating that mGluR5 is necessary for the full expression of VMR in response to bladder distention in the absence of inflammation. Furthermore, we observed that mice infected with a uropathogenic strain of *Escherichia coli *(UPEC) develop inflammatory hyperalgesia to bladder distention, and that the selective mGluR5 antagonist fenobam [N-(3-chlorophenyl)-N'-(4,5-dihydro-1-methyl-4-oxo-1H-imidazole-2-yl) urea], reduces the VMR to bladder distention in UPEC-infected mice.

**Conclusions:**

Taken together, these data suggest that mGluR5 modulates both inflammatory and non-inflammatory bladder nociception, and highlight the therapeutic potential for mGluR5 antagonists in the alleviation of bladder pain.

## Background

Interstitial cystitis/painful bladder syndrome (IC/PBS) is a serious and painful condition of unknown etiology that affects 3-6% of women in the United States [1,2]. The major clinical symptom of IC/PBS is pain upon bladder filling (distention) leading to urinary frequency and urinary urgency [3]. The current available treatments are often ineffective and do not treat the underlying pathology. Rodent bladder-injury models that induce some of the symptoms observed in IC/PBS have been used to evaluate potential treatments for IC/PBS [4-9]. One injury model, bacterial cystitis (urinary tract infection, UTI) is known to cause a similar constellation of symptoms as observed in IC/PBS (i.e. urinary frequency and urgency [10-12]). In addition, bacterial cystitis can be modeled in rodents through bladder exposure to uropathogenic *Escherichia Coli *(UPEC) [13,14]. Bladder infections due to UPEC are responsible for approximately 80% of UTIs in otherwise healthy women [15,16]. Understanding the underlying molecular mechanisms of both non-inflammatory bladder pain and inflammatory bladder pain due to UPEC infection could lead to the development of novel treatments for painful bladder infections as well as for IC/PBS and possibly other visceral pain conditions.

Glutamate is the predominant excitatory neurotransmitter in the mammalian nervous system [17-19]. Glutamate mediates its effects through two major classes of glutamate receptors: ligand-gated ionotropic receptors (iGluRs) and G protein-coupled metabotropic glutamate receptors (mGluRs). Among the metabotropic glutamate receptors, one subtype, mGluR5, is of particular interest in the context of pain conditions. mGluR5 is expressed throughout the peripheral and central nervous system [20] and has previously been shown to have a pro-nociceptive role in a variety of somatic pain models [20-25] and some visceral pain models [26-28]. Specific to visceral pain models, mGluR5 was found to modulate gastroesophogeal and colorectal afferent sensitivity [26,27,29]. Based on this prior information, a previous study examined the ability of the mGluR5 antagonist, MPEP (2-methyl-6-(phenylethynyl)-pyridine), to reduce bladder pain responses in naïve (uninjured) rats [30]. While this study suggests a potential role for mGluR5 in bladder pain, the evidence is based exclusively on the use of MPEP, which has recently been shown to act non-selectively *in vivo *[31]. Thus, these intriguing initial findings are in need of validation. Furthermore, the role of mGluR5 in inflammatory bladder pain is unknown. Here, using a combination of genetic and pharmacological approaches we demonstrate that mGluR5 regulates both bladder nociception and normal bladder function in naïve mice. Furthermore, we observed an increased VMR to bladder distention in mice infected with UPEC. Finally, UPEC-induced hyperalgesia is reduced by treatment with the specific mGluR5 antagonist, fenobam. Together these data strongly support the hypothesis that mGluR5 is necessary for the full expression of inflammatory and non-inflammatory bladder nociception and may be a relevant target for the treatment of bladder pain arising from multiple pathologies, including IC/PBS.

## Results

### mGluR5 is necessary for the full expression of non-inflammatory bladder nociception

**Figure 1:**
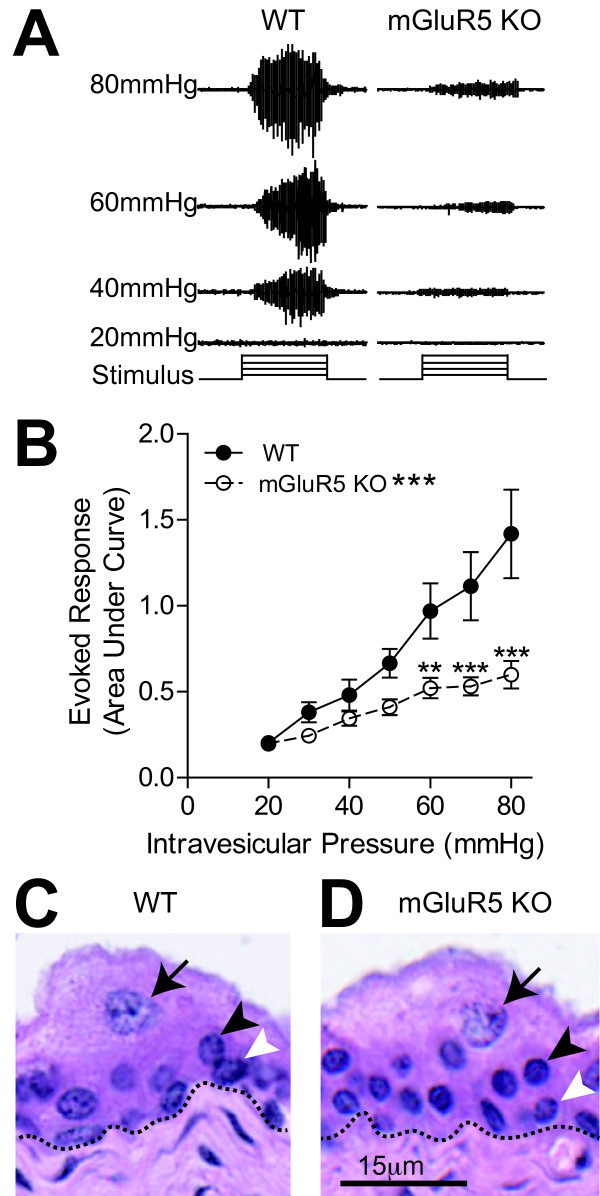
**Reduced visceromotor response to bladder distension in mGluR5 knockout mice compared to wild type littermates**. **A**. Representative VMR tracings from a WT and mGluR5 KO mice. As the intravesicular pressure is increased (20-80 mmHg), the EMG activity of the abdominal muscle (VMR) is also increased. The total amount of activity (area under the curve) during the 20 second distention is calculated to determine the evoked response at each pressure. **B**. mGluR5 KO (*n *= 18) mice have a significantly blunted VMR when compared to WT littermates (*n *= 15) +/- SEM,* *p *< 0.05, **p < 0.01, ***p < 0.001. 2-way ANOVA with Bonferroni post-hoc test. There were no obvious histological differences observed between mGluR5 KO mice (1D) and their WT littermates (1C). In both, the urothelium (above dashed line) has normal layers of superficial facet cells (arrows), intermediate cells (black arrowhead) and basal cells (white arrowhead).

To assess bladder nociception in response to distension, we utilized the distension-evoked visceromotor response (VMR). The VMR is a spinobulbospinal reflex to bladder distention, increased in decerebrate mice/rats and absent in mice/rats with an acute mid thoracic spinal cord transection [32-34]. Bladder distention reliably produces pain and/or discomfort in humans [35], and is frequently used in rodents as a visceral pain model [5,30,33]. To provide genetic evidence supporting a role for mGluR5 in bladder nociception, we tested the VMR to bladder distention in mGluR5 knockout mice (mGluR5 KO) compared to their WT littermates. Stepwise increases in bladder distension resulted in progressively larger VMR in wild type mice, as shown in Figure 1B. Furthermore, mGluR5 KO mice showed a statistically significant decrease in the evoked response to bladder distention (VMR) in the noxious range of pressures when compared to the VMR of WT littermates (p < 0.0001).

There are several possible reasons that genetic ablation of mGluR5 could lead to the observed suppression of the VMR. Recent work has implicated the urothelium in the sensation of physical and chemical stimuli [36]. One possibility is that loss of mGluR5 could lead to anatomical changes in the bladder lining, thus altering the response to distension. We therefore examined histological sections from mGluR5 KO mice and their WT littermates. The absence of mGluR5 does not appear to impact bladder architecture, as the bladders from mGluR5 KO mice are histologically indistinguishable from WT littermates. The superficial, intermediate and basal epithelial cells are present (see Figure 1C and 1D) and the underlying mesenchyme and muscle layers also remain intact. Furthermore, the barrier formed by the superficial cells is normal as evidenced by Uroplakin III staining (data not shown) in both the WT and mGluR5 KO mice. Therefore, the absence of mGluR5 affects the response to noxious bladder distention but does not alter gross urothelial architecture.

**Figure 2:**
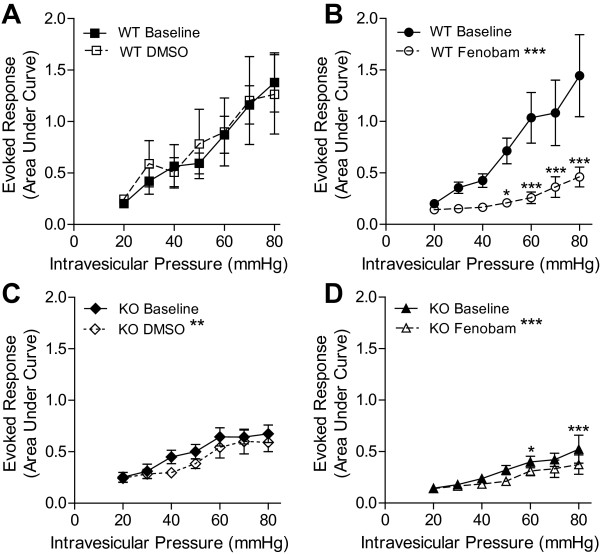
**The selective mGluR5 antagonist, fenobam, is analgesic in distention-induced bladder pain model**. **A**. Treatment with the vehicle used to dissolve fenobam (100% DMSO) has no effect on the response to bladder distention. **B**. Treatment with an mGluR5 antagonist, fenobam, is analgesic in a bladder distention-evoked pain model. C&D. Fenobam and DMSO reduce the evoked response in mGluR5 KO mice. +/- SEM,* *p *< 0.05, **p < 0.01, ***p < 0.001. 2-way ANOVA with Bonferroni post-hoc test.

Compensatory changes in gene expression represent a potential confound to any experiment utilizing genetic manipulation. Thus, the robust phenotype observed in the mGluR5 KO mice could be the result of compensatory expression changes in other genes that have a role in bladder nociception. An acute pharmacological blockade of the receptor represents a potentially powerful approach to complement these findings from genetically modified mice. However, as mentioned above, the pharmacologic agent (MPEP) used in prior studies shows a clear lack of specificity in vivo [31]. To ask whether mGluR5 activation is acutely involved in distention-induced bladder nociception, we tested whether specific pharmacologic inhibition of mGluR5 with the selective antagonist fenobam [31,37,38], would suppress the VMR to urinary bladder distention. Systemic (intraperitoneal (IP)) administration of fenobam to WT mice resulted in a statistically significant reduction in the response to bladder distention compared to pretreatment baseline responses (Figure 2B, *p *< 0.0001), whereas treatment with vehicle had no statistically significant effect on VMR compared to baseline (Figure 2A). Thus, pretreatment with fenobam mimicked the reduced nociceptive response to bladder distention that was observed in mGluR5 KO mice relative to their WT littermates (compare Figure 1A and 2A). We next evaluated the effect of fenobam on the VMR of mGluR5 KO mice. Surprisingly, treatment with either vehicle (DMSO) or fenobam caused a small but statistically significant decrease in the VMR evoked by bladder distention compared to baseline responses in mGluR5 KO mice (Figure 2C and 2D, *p *< 0.001 and *p *= 0.007).

### mGluR5 regulates intermicturition interval (IMI)

**Figure 3:**
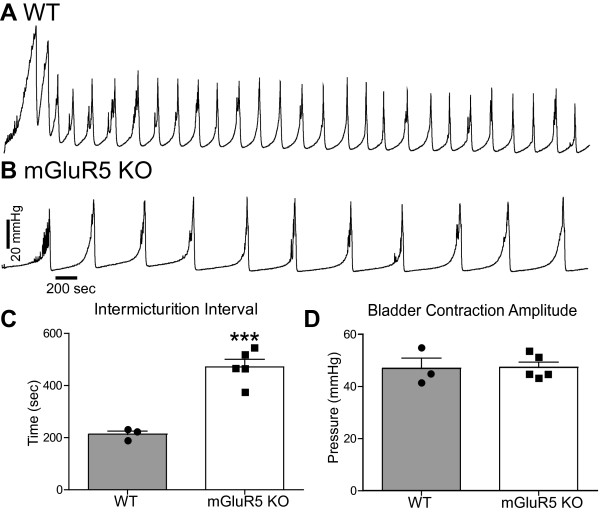
**mGluR5 KO mice have an increased intermicturition interval**. **A**. Representative urodynamic profile of a WT mouse. **B**. Representative urodynamic profile of a mGluR5 KO mouse. A-C. The IMI in WT mice was significantly smaller when compared to mGluR5 KO mice (WT baseline IMI 212.3 ± 12.94 *N *= 3, mGluR5 KO IMI baseline 471.5 ± 29.14 *N *= 5). **D**. However, there was no difference in the bladder contraction amplitude. ***P < 0.001 unpaired Student's t-test compared to WT IMI.

To test the role of mGluR5 in urodynamics, we compared the cystometry profile of mGluR5 KO mice and their WT littermates. mGluR5 KO mice had a significantly increased IMI (212.3 seconds *versus *471.5 seconds, for WT and KO, respectively, *p *= 0.0006) (Figure 3A-C). Despite the difference in IMI, the average amplitude of bladder contractions was not significantly different in mGluR5 KO mice relative to their WT littermates (Figure 3D, *p *= 0.9215).

**Figure 4:**
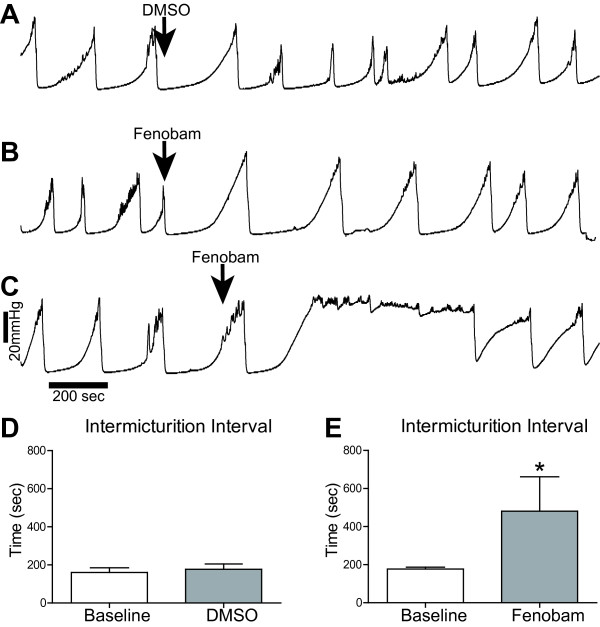
**Fenobam treatment increases the intermicturition interval**. **A**-**C**. Representative urodynamic profile of WT mice before and after treatment. An intraperitoneal (IP) injection with fenobam significantly increased the IMI in 4/8 mice treated (**B**, **E**), while 4/8 mice stopped cycling (**C**). In the mice that stopped cycling, the mean time to resume bladder cycling and micturition after IP fenobam administration was 18.6 ± 5.0 minutes. The IMI was not significantly affected by IP DMSO (*n *= 7) (**A**, **D**). *P < 0.05 paired Student's t-test compared to baseline.

To determine if the voiding behavior we observed in mGluR5 KO mice was the result of genetic ablation of mGluR5 or the result of compensatory changes, we acutely treated WT mice with fenobam or vehicle and measured the effect on urodynamics. While vehicle treatment had no effect on IMI (160.2 seconds versus 177.1 seconds, for baseline and vehicle (DMSO) respectively, *p *= 0.66, Figure 4D), fenobam treatment increased the IMI when compared to the baseline IMI. The IMI before fenobam treatment was 182 seconds (*n *= 8). After IP fenobam treatment, four of the eight mice stopped bladder cycling and micturition immediately (Figure 4C), while four of the mice had significantly increased IMI (481.3 seconds, *p *= 0.031, Figure 4D and 4E). The mean time to resume bladder cycling and micturition after systemic fenobam administration was 18.6 minutes. This suggests a tight coupling between mGluR5 activation and micturition cycling. The acute effect of the mGluR5 antagonist also suggests that the increased IMI observed in mGluR5 KO mice is not likely due to developmental changes as a result of genetic ablation of mGluR5.

### UPEC infection results in changes in bladder histology and an increased VMR

**Figure 5:**
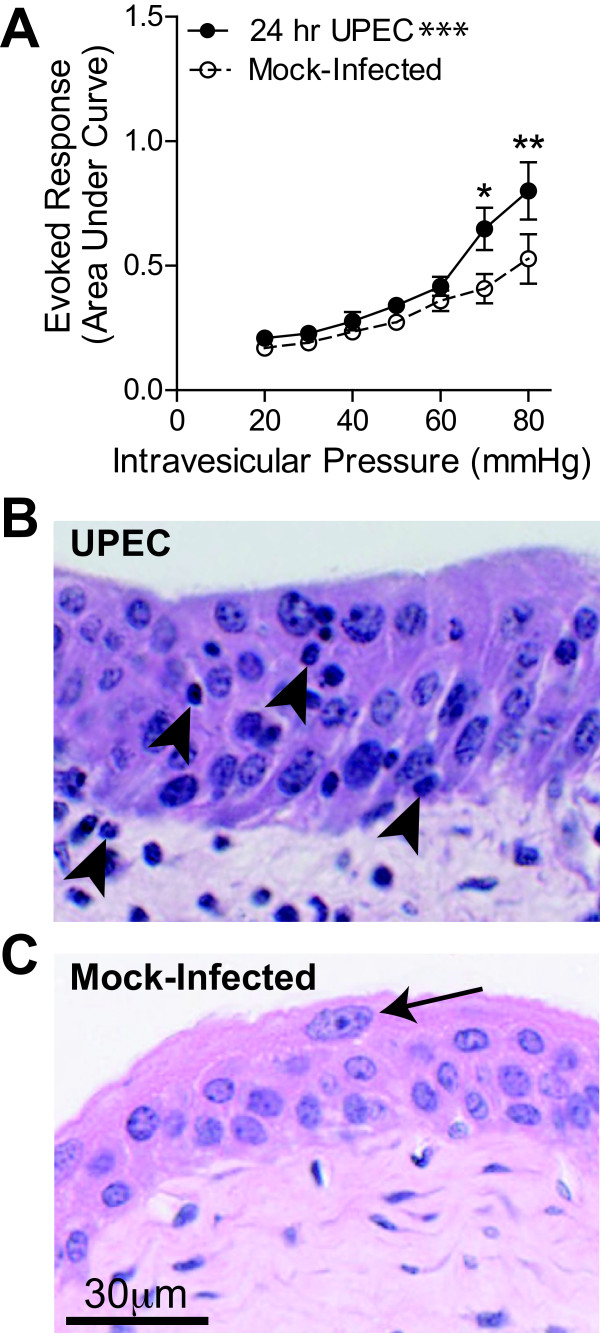
**UPEC Infection results in histological changes and bladder hyperalgesia**. **A**. 24 hour infection with uropathogenic E. coli (UPEC) increases the VMR to bladder distention when compared to mock-infected (PBS) littermates. **B**. Infection with UPEC results in an increased infiltration of polymorphonuclear leukocytes (arrowheads), and a sloughing of the superficial facet cells. **C**. Distention alone does not result in the loss of superficial facet cells (arrows,). +/- SEM *p < 0.05, **p < 0.01, ***p < 0.001. Unpaired 2-way ANOVA with Bonferroni post-hoc test.

Our results, together with previous studies [30,39,40] suggest that mGluR5 is involved in the mediation of micturition cycling and controls nociceptive responses during noxious bladder distention; however, the role of mGluR5 in inflammatory bladder nociception is unexplored. We therefore used the UPEC model of bladder inflammation to examine the role of mGluR5 in inflammatory bladder nociception. We found that UPEC infection resulted in an increased VMR to noxious bladder distention when compared to mock-infected littermate controls (Figure 5A, *p *< 0.0001). Furthermore, in mice whose bladders had been distended, UPEC infection produced an increased infiltration of polymorphonuclear leukocytes observed throughout the full thickness of the bladder tissue (Figure 5B, arrowheads), as well as an increased tissue thickness of both the urothelium and mesenchyme compared to distended mock-infected mice (Figure 5C). Noxious bladder distention combined with UPEC infection resulted in greater histological damage as measured by tissue inflammation scores when compared to mock-infected mice with noxious distention (data not shown) [41]. UPEC infection (alone and with distention) resulted in barrier disruption through a loss of superficial facet cells, while noxious distention alone had no effect on these cells and the barrier remained intact (arrows, Figure 5C).

### mGluR5 is necessary for the full expression of inflammatory bladder nociception

**Figure 6:**
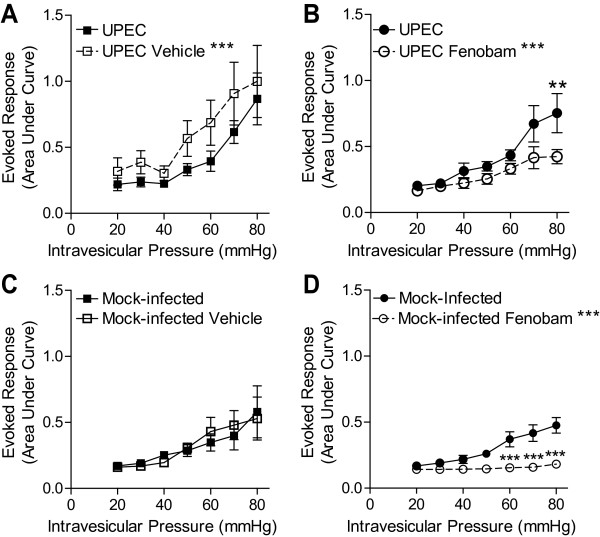
**Treatment with an mGluR5 antagonist, fenobam, is analgesic in a UPEC infection-induced inflammatory bladder distention-evoked pain model**. Treatment with the vehicle used to dissolve fenobam (100% DMSO) increases the evoked response to bladder distention (A, *n *= 5), whereas treatment with fenobam significantly reduced the evoked response to bladder distension (B, *n *= 7). In mock-infected animals, treatment with the vehicle used to dissolve fenobam (100% DMSO) had no effect on the evoked response (C, *n *= 6), whereas fenobam significantly reduced the evoked response (D, *n *= 6). * *p *< 0.05, **p < 0.01, ***p < 0.001. Paired 2-way ANOVA with Bonferroni post-hoc test

In mice with a UPEC-induced UTI, mice treated with vehicle had an increased VMR when compared to baseline (Figure 6A, *p *= 0.0007). While vehicle treatment increased the VMR, treatment with fenobam resulted in a significantly reduced VMR when compared to pre-fenobam measurements (Figure 6B, *p *= 0.0006). In contrast, vehicle had no effect on the VMR in mice mock infected (with PBS instead of UPEC, Figure 6C). In these control mice (mock infected), treatment with fenobam significantly reduced the VMR when compared to pre-treatment VMR measurements (Figure 6D). These results are consistent with those observed in WT mice (not mock-infected, Figures **Error! Reference source not found**. A and 2B).

## Discussion

Clinically, visceral pain is often treated as a variant of somatic pain under the assumption that one neurological mechanism may be responsible for both visceral and somatic pain [42]. However, conventional treatments for somatic pain are often ineffective in treating visceral pain, indicating significant differences between visceral and somatic pain [42,43]. This is likely due, in part, to intrinsic differences between visceral nociceptors and non-visceral nociceptors (Reviewed in: [43-45]). Our results support this difference as we found that blocking mGluR5 activation resulted in a decreased VMR in the absence of injury. Whereas previous work has demonstrated that blocking mGluR5 activation has no effect on somatic pain in the absence of injury [31,46].

Although the role of mGluR5 in inflammatory bladder pain is unclear, a few reports have shown that mGluR5 antagonism can lead to analgesia in visceral pain models (acetic acid writhing and colonic distention) [23,24,27,30,37,47-49]. A recent publication reported that an mGluR5 antagonist, MPEP, effectively reduced the nociceptive VMR to noxious distention of non-inflamed bladders in rats [30]. However, MPEP, has since been shown to have off-target effects, as it retains analgesic efficacy in mGluR5 KO mice [31]. Thus, the precise role of mGluR5 in bladder distention-induced nociception remains unclear. Further, it is unknown whether mGluR5 activation has a role in the more clinically relevant condition of inflammatory bladder pain. In the present study, we demonstrate that mGluR5 is necessary for the full expression of distention-induced bladder nociception in naïve as well as UPEC-infected mice.

Here, we demonstrate the importance of mGluR5 in non-inflammatory bladder nociception using both genetic and pharmacologic techniques. First, mice lacking mGluR5 have a significantly reduced response to noxious bladder distention when compared to their WT littermates. Despite this dramatic effect, mGluR5 KO mice have no baseline motor deficiencies [50]. However, genetic deletion of mGluR5 could result in unpredictable upregulation or compensation of other genes involved in bladder function. Acute systemic treatment with a selective mGluR5 antagonist (fenobam) mimics the blunted VMR observed in the mGluR5 KO mice, suggesting that the absence of mGluR5 activity accounts for the differences in VMR as well as the cystometric profile observed in mGluR5 KO mice. Together these data suggest that mGluR5 is necessary for the full expression of distention-induced bladder pain in naïve mice and highlight the therapeutic potential for mGluR5 antagonists in the treatment of bladder pain.

Surprisingly, both fenobam and vehicle (DMSO) induced a small but statistically significant reduction in the distention induced VMR in mGluR5 KO mice. Fenobam at the dose used in this study was previously demonstrated to be specific to mGluR5 *in vivo *[31], and we do not believe that these results challenge that finding because fenobam but not DMSO, had an effect on the VMR of WT mice. Because both fenobam and vehicle significantly reduced the VMR in mGluR5 KO mice, we conclude that the vehicle is having effects in the KO that are not observed in WT mice. A possible explanation for the effects of the DMSO in KO but not in WT mice is that mGluR5 KO mice are significantly smaller than their WT littermates [51]. DMSO has been shown to block peripheral C-fiber nerve conduction [52]. It is possible that the volume (20 μl) delivered in relation to the weight of the mGluR5 KO mice leads to such a conduction block.

The dramatic phenotype of mGluR5 KO mice as well as the significantly reduced pain response in fenobam-treated WT mice suggests that mGluR5 regulates bladder nociception. It is also possible that mGluR5 could regulate normal bladder function such as micturition. To examine the role of mGluR5 in normal bladder function, we examined the cystometric profiles of mGluR5 KO mice compared to their WT littermates. Genetic disruption of mGluR5 significantly increased the IMI, but had no effect on the bladder contraction amplitude (pressure at which voiding occurred). An increased IMI suggests that mice lacking mGluR5 are able to urinate, but they tolerate higher bladder volumes (*i.e*. higher bladder distension) before voiding. Our results are consistent with previous work demonstrating a role for mGluR5 in the micturition reflex [30,39,40]. Importantly, mGluR5 does not appear to be involved in bladder development, as bladders of mGluR5 KO mice were indistinguishable from their WT littermates upon gross inspection. These results suggest that mGluR5 has a role in bladder sensation in naïve mice.

Bladder infections due to UPEC are the most common reason that women see a doctor [11,53]. This is the first evidence that infection with UPEC sensitizes mice to noxious bladder distention. WT mice infected with UPEC had a greater VMR to bladder distention when compared to mock-infected littermate controls. These data are consistent with the finding that mice with a UTI have increased referred abdominal pain [54]. Furthermore, our results indicate that pharmacologic blockade of mGluR5 activation can reduce the VMR to bladder distention in UPEC-infected mice. The reduction in the distention-evoked VMR after fenobam treatment in mice infected with UPEC may be even larger than is apparent in Figure 6B, as the VMR is actually increased in vehicle-treated mice (see Figure 6A). Because distention in combination with UPEC infection was more damaging to bladder histology than distention in mock infected mice, the increased VMR after vehicle is likely the result of injury induced by bladder infection in combination with repeated noxious bladder distention. Another, yet unlikely, explanation is that systemic DMSO might lead to bladder sensitization in mice with UPEC infection.

In models of somatic inflammatory pain, an increase in mGluR5 expression was observed in the CNS, specifically the central nucleus of the amygdala and the spinal cord [55,56]. Whether mGluR5 expression is altered in the context of visceral inflammatory pain remains to be determined.

## Conclusions

Up to 85% of community-acquired UTIs are due to infection with UPEC [57,58]. Standard-of-care for a UTI is antibiotic treatment. However, in women with normal urological anatomy (uncomplicated UTI), UTIs are self-limiting [16]. Therefore, uncomplicated UTIs will resolve without antibiotic treatment. Despite this, the pain due to a UTI necessitates antibiotic treatment for symptomatic relief. Antibiotic use can lead to UTI recurrence by selecting for antibiotic resistant bacteria [16]. By treating the pain of a UTI rather than the infection, it may be possible to allow the UTI to take its natural course and become self-limited and reduce antibiotic-resistant reoccurrence. Based on our findings, we suggest that mGluR5 antagonists could be a useful for therapy for symptomology in UTI that could reduce antibiotic use.

Although less common, bladder pain in the form of IC/PBS is a serious and debilitating medical condition. Unfortunately for patients, no reliably effective treatment options exist. Previous work has suggested a role for mGluR5 in the CNS in distention-induced bladder pain [30]. However, the mGluR5 antagonist used in this study (MPEP) is analgesic in mGluR5 KO mice, suggesting off-target effects [31]. Here, using a combination of genetics and pharmacology, we demonstrate that mGluR5 has a crucial role in the modulation of distention induced bladder nociceptive responses. Because in these studies mGluR5 was blocked systemically and knocked out globally, the anatomical localization where mGluR5 modulates bladder pain is undefined. In addition, mGluR5 is expressed throughout the peripheral and central nervous system [20,21,46,59-62]. However, it should be noted that the mGluR5 antagonist, fenobam, readily and rapidly crosses the blood brain barrier [31]. If the chronic pain experienced due to IC/PBS is also mediated by mGluR5, then antagonists of mGluR5 such as fenobam may be useful in treating chronic bladder pain. Antagonists of mGluR5 may have clinical potential for the treatment of both acute and chronic bladder pain. Previous work using non-visceral pain models has demonstrated that mGluR5 activity modulates the activation of ERK1/2 (extracellular regulated kinase 1/2) in both the spinal cord [25,63] and/or central nucleus of the amygdala (CeA) [46,64]. Inhibition of mGluR5 in the CeA is analgesic in multiple models of somatic inflammatory pain [46,60]. Furthermore, recent work from our lab has demonstrated that inhibition of spinal ERK signaling via intrathecally administered MEK inhibitors is analgesic in both inflammatory and non-inflammatory bladder pain models [5]. Future work can help determine the anatomical and molecular mechanisms by which mGluR5 modulates bladder nociceptive responses.

## Methods

1. Subjects and ethical approval

All animal experiments were performed in accordance with the guidelines of the Committee for research and Ethical Issues of International Association for the Study of Pain [65]. The experimental protocol was approved by the Washington University Institutional Animal Care and Use Committee (St. Louis, MO). All experiments were performed on female mice aged 10-13 weeks. All mice were humanely euthanized at the end of the experiments by decapitation under deep isoflurane (5%) anesthesia. Female C57BL/6 mice used in UPEC experiments were purchased from The Jackson Laboratory (Bar Harbor, ME). For experiments involving mice lacking mGluR5 (mGluR5 KO), animals were bred in-house on a C57BL/6 background and compared with wildtype (WT) littermates [50]. Unless otherwise indicated, all mice were group housed in cages of 3-5. All mice were kept on a 12:12-h light/dark schedule with ad libitum access to food and water.

2. Drugs and other agents

Fenobam was purchased from Tocris Bioscience (Ellisville, MO), and is a specific negative allosteric modulator of mGluR5 [31,38]. The dosage (30 mg/kg, intraperitoneal) of fenobam was chosen because it was the lowest effective dose to induce analgesia in two mouse models of inflammatory pain, and had no effect on mGluR5 KO mice [31]. Fenobam was dissolved in 100% dimethyl sulfoxide (DMSO, Sigma-Aldrich, St. Louis, MO) on the day of the experiment. All intraperitoneal injections were 20 μl. Throughout all experiments and analysis, the investigator performing the VMR was blinded to pharmacological treatment.

3. Phasic bladder distentions and VMR measurements

Visceral nociception was quantified using VMR, an electromyographic (EMG) recording of the abdominal muscle response to bladder distention that has previously been validated as a measure of nociception [34,66]. An example of the bladder-distention induced VMR of WT mice can be seen in Figure 1. Abdominal VMRs to urinary bladder distention were performed as described previously [5]. The mice were anesthetized with 2% isoflurane, and chlorinated silver wire electrodes were placed on the superior oblique abdominal muscle. One was placed subcutaneously across the abdominal wall (as a ground) to allow differential amplification of the abdominal VMR signals. A lubricated 24G angiocatheter was inserted into the bladder via the urethra for bladder distention. After completion of the surgical preparation, isoflurane anesthesia was reduced to approximately 0.9% until a flexion reflex response was present (evoked by pinching the paw) but spontaneous escape behavior and righting reflex were absent. Once a stable depth of anesthesia was obtained, the level was not changed for the duration of the experiment. The animals were not restrained in any fashion. Body temperature was monitored throughout the experiment and maintained using an overhead radiant light. Phasic bladder distention with compressed air was then used to evoke a VMR. The air pressure was controlled by an automated distention control device custom made in the Washington University School of Medicine Electronics Shop. The distention stimulus applied 20 to 80 mm Hg pressure (10 mmHg steps) for 20 seconds every 2 minutes. The VMR signal was relayed in real time using a Grass CP511 preamplifier (Grass Technologies, West Warwick, RI) to a PC via WinDaq DI-720 module (Dataq Instruments, Arkon, OH). These data were exported to Igor Pro 6.05 software (Wavemetrics, Portland, OR). Using a custom script, the VMR signals were subtracted from the baseline, rectified, and integrated over 20 seconds to quantify the area under the curve (see Figure 1). The VMR is presented in arbitrary units. After baseline responses to distensions 20 mmHg-80 mmHG (10 mmHg steps) were recorded, the mouse was allowed to recover for at least 30 min (level of anesthesia remained unchanged). Following baseline recordings, the mice were given a 20 ul IP injection of either fenobam (30 mg/kg, dissolved in 100% DMSO) or 100% DMSO (vehicle). A second set of bladder distentions (20 mmHg-80 mmHg, 10 mmHg steps) was started 5 minutes following administration of fenobam or vehicle and completed within 45 minutes. The investigator who tested, processed and quantified the VMR was blinded to the drug treatment and genotype.

4. Infection with UPEC

Infection with a clinical UPEC isolate, UTI89, was performed as previously described [67]. Briefly, mice were anesthetized with isoflurane and inoculated via transurethral catheterization with 50 μl of 2-8 × 10^7 ^colony-forming units/mL of bacteria in phosphate buffered saline (PBS). Control mice (mock-infection) received 50 ul of intravesicular PBS. Following intravesicular instillation of either PBS or UPEC, the mice were individually housed. Urine was collected at 6 and 24 hours, and infection was confirmed. At 24 hours post infection, the VMR response to bladder distention was recorded as previously described [5]. Following the VMR, isoflurane anesthesia was increased and the mice were euthanized by cervical dislocation. Bladder tissue was quickly removed and fixed in methacarn (60% methanol, 30% chloroform, 10% glacial acetic acid) [13]. The bladder tissue was then embedded in paraffin, sectioned and stained with hemotoxylin and eosin. Photomicrographs were taken using a Hamamatsu NanoZoomer HT (Hamamatsu Corporation).

5. Urodynamics

Urodynamic recordings were performed as described previously [68]. Briefly, mice were anesthetized with subcutaneous urethane (1.2 g/kg). A midline laparotomy incision was made, and the dome of the urinary bladder was exposed. The bladder dome was punctured with a 25-gauge needle. Intravesicular pressure was monitored continuously *in vivo *while the bladder was filled with room temperature saline at a rate of 0.04 mL per minute using a syringe pump (KD Scientific, New Hope, PA). Voiding was allowed to occur spontaneously via the urethra. The intravesicular pressure was recorded in real time using WINDAQ data acquisition program (DataQ Instruments, Akron, OH) at a sampling rate of 20 Hz. The intermicturition interval (IMI) was calculated as the average time (seconds) between peaks. The amplitude of contraction was the value (cmHg) of the peak.

## Abbreviations

DMSO: Dimethyl sulfoxide; IC/PBS: Interstitial cystitis/painful bladder syndrome; IMI: Intermicturition interval; IP: Intraperitoneal; KO: Knockout; mGluRs: Metabotropic glutamate receptors; mGluR5: Metabotropic glutamate receptor 5; MPEP: 2-methyl-6-(phenylethynyl)-pyridine; PBS: Phosphate-buffered saline; UPEC: Uropathogenic *Escherichia Coli*; UTI: Urinary tract infection; VMR: Visceromotor response; WT: Wildtype

## Competing interests

The authors declare that they have no competing interests.

## Authors' contributions

LWC, KMS, DGS, PA, CSQ and SKV performed experiments and analyzed data. LWC, KMS, SKV, HHL, IUM and RWG conceived the study and contributed to writing the manuscript. All authors read and agreed on the final version of the manuscript. KMS and LWC contributed equally to this manuscript. KMS created the cover art figure. All Authors read and approved the final manuscript.

## References

[B1] Rosenberg MT Page S Hazzard MA Prevalence of interstitial cystitis in a primary care setting Urology 2007 69 48 52 10.1016/j.urology.2006.03.085 17462479

[B2] Berry SH Elliott MN Suttorp M Bogart LM Stoto MA Eggers P Nyberg L Clemens JQ Prevalence of symptoms of bladder pain syndrome/interstitial cystitis among adult females in the united states J Urol 2011 186 540 544 10.1016/j.juro.2011.03.132 21683389 PMC3513327

[B3] Warren JW Brown J Tracy JK Langenberg P Wesselmann U Greenberg P Evidence-based criteria for pain of interstitial cystitis/painful bladder syndrome in women Urology 2008 71 444 448 10.1016/j.urology.2007.10.062 18342184 PMC2293273

[B4] Maggi CA Lecci A Santicioli P Del Bianco E Giuliani S Cyclophosphamide-induced cystitis in rats: involvement of capsaicin-sensitive primary afferents Agents Actions 1993 38 Spec No C28 30 8317316 10.1007/BF01991127

[B5] Lai HH Qiu CS Crock LW Morales ME Ness TJ Gereau RW Activation of spinal extracellular signal-regulated kinases (ERK) 1/2 is associated with the development of visceral hyperalgesia of the bladder Pain 2011 152 2117 2124 10.1016/j.pain.2011.05.017 21705143 PMC3157542

[B6] Westropp JL Buffington CA In vivo models of interstitial cystitis J Urol 2002 167 694 702 10.1016/S0022-5347(01)69129-8 11792956

[B7] Saban MR Saban R Hammond TG Haak-Frendscho M Steinberg H Tengowski MW Bjorling DE LPS-sensory peptide communication in experimental cystitis Am J Physiol Renal Physiol 2002 282 F202 F210 11788433 10.1152/ajprenal.0163.2001

[B8] Boudes M Uvin P Kerselaers S Vennekens R Voets T De Ridder D Functional characterization of a chronic cyclophosphamide-induced overactive bladder model in mice Neurourol Urodyn 2011 30 1659 1665 10.1002/nau.21180 21717507

[B9] Dang K Lamb K Cohen M Bielefeldt K Gebhart GF Cyclophosphamide-induced bladder inflammation sensitizes and enhances P2X receptor function in rat bladder sensory neurons J Neurophysiol 2008 99 49 59 17959738 10.1152/jn.00211.2007 PMC2659400

[B10] Bower JM Eto DS Mulvey MA Covert operations of uropathogenic Escherichia coli within the urinary tract Traffic 2005 6 18 31 10.1111/j.1600-0854.2004.00251.x 15569242 PMC2523259

[B11] Baerheim A Hunskar S Current management of uncomplicated acute cystitis in general practice Tidsskr Nor Laegeforen 1997 117 1304 1307 9182361

[B12] Malterud K Baerheim A Peeing barbed wire Symptom experiences in women with lower urinary tract infection Scand J Prim Health Care 1999 17 49 53 10.1080/028134399750002908 10229994

[B13] Mysorekar IU Hultgren SJ Mechanisms of uropathogenic Escherichia coli persistence and eradication from the urinary tract Proc Natl Acad Sci USA 2006 103 14170 14175 10.1073/pnas.0602136103 16968784 PMC1564066

[B14] Mulvey MA Lopez-Boado YS Wilson CL Roth R Parks WC Heuser J Hultgren SJ Induction and evasion of host defenses by type 1-piliated uropathogenic Escherichia coli Science (New York, NY 1998 282 1494 1497 10.1126/science.282.5393.1494 9822381

[B15] Foxman B Epidemiology of urinary tract infections: incidence, morbidity, and economic costs Am J Med 2002 113 Suppl 1A 5 13 10.1016/s0002-9343(02)01054-9 12113866

[B16] Foxman B The epidemiology of urinary tract infection Nat Rev Urol 2010 7 653 660 10.1038/nrurol.2010.190 21139641

[B17] Matsumoto G Hisamitsu T de Groat WC Role of glutamate and NMDA receptors in the descending limb of the spinobulbospinal micturition reflex pathway of the rat Neurosci Lett 1995 183 58 61 10.1016/0304-3940(94)11114-X 7746488

[B18] Yoshiyama M de Groat WC Supraspinal and spinal alpha-amino-3-hydroxy-5-methylisoxazole-4-propionic acid and N-methyl-D-aspartate glutamatergic control of the micturition reflex in the urethane-anesthetized rat Neuroscience 2005 132 1017 1026 10.1016/j.neuroscience.2005.01.041 15857706 PMC3118677

[B19] Kakizaki H Yoshiyama M Roppolo JR Booth AM De Groat WC Role of spinal glutamatergic transmission in the ascending limb of the micturition reflex pathway in the rat J Pharmacol Exp Ther 1998 285 22 27 9535990

[B20] Varney MA Gereau RW Metabotropic glutamate receptor involvement in models of acute and persistent pain: prospects for the development of novel analgesics Curr Drug Targets CNS Neurol Disord 2002 1 283 296 10.2174/1568007023339300 12769620

[B21] Karim F Bhave G Gereau RW Metabotropic glutamate receptors on peripheral sensory neuron terminals as targets for the development of novel analgesics Mol Psychiatry 2001 6 615 617 10.1038/sj.mp.4000961 11673787

[B22] Bhave G Karim F Carlton SM Gereau RW Peripheral group I metabotropic glutamate receptors modulate nociception in mice Nat Neurosci 2001 4 417 423 10.1038/86075 11276233

[B23] Walker K Bowes M Panesar M Davis A Gentry C Kesingland A Gasparini F Spooren W Stoehr N Pagano A Metabotropic glutamate receptor subtype 5 (mGlu5) and nociceptive function. I. Selective blockade of mGlu5 receptors in models of acute, persistent and chronic pain Neuropharmacology 2001 40 1 9 10.1016/S0028-3908(00)00113-1 11077065

[B24] Hudson LJ Bevan S McNair K Gentry C Fox A Kuhn R Winter J Metabotropic glutamate receptor 5 upregulation in A-fibers after spinal nerve injury: 2-methyl-6-(phenylethynyl)-pyridine (MPEP) reverses the induced thermal hyperalgesia J Neurosci 2002 22 2660 2668 11923431 10.1523/JNEUROSCI.22-07-02660.2002 PMC6758316

[B25] Hu HJ Alter BJ Carrasquillo Y Qiu CS Gereau RW Metabotropic glutamate receptor 5 modulates nociceptive plasticity via extracellular signal-regulated kinase-Kv4.2 signaling in spinal cord dorsal horn neurons J Neurosci 2007 27 13181 13191 10.1523/JNEUROSCI.0269-07.2007 18045912 PMC6673402

[B26] Young RL Page AJ O'Donnell TA Cooper NJ Blackshaw LA Peripheral versus central modulation of gastric vagal pathways by metabotropic glutamate receptor 5 Am J Physiol Gastrointest Liver Physiol 2007 292 G501 G511 17053158 10.1152/ajpgi.00353.2006

[B27] Lindstrom E Brusberg M Hughes PA Martin CM Brierley SM Phillis BD Martinsson R Abrahamsson C Larsson H Martinez V Blackshaw LA Involvement of metabotropic glutamate 5 receptor in visceral pain Pain 2008 137 295 305 10.1016/j.pain.2007.09.008 17937975

[B28] Bianchi R Rezzani R Borsani E Rodella L mGlu5 receptor antagonist decreases Fos expression in spinal neurons after noxious visceral stimulation Brain Res 2003 960 263 266 10.1016/S0006-8993(02)03697-1 12505681

[B29] Page AJ Young RL Martin CM Umaerus M O'Donnell TA Cooper NJ Coldwell JR Hulander M Mattsson JP Lehmann A Blackshaw LA Metabotropic glutamate receptors inhibit mechanosensitivity in vagal sensory neurons Gastroenterology 2005 128 402 410 10.1053/j.gastro.2004.11.062 15685551

[B30] Hu Y Dong L Sun B Guillon MA Burbach LR Nunn PA Liu X Vilenski O Ford AP Zhong Y Rong W The role of metabotropic glutamate receptor mGlu5 in control of micturition and bladder nociception Neurosci Lett 2009 450 12 17 10.1016/j.neulet.2008.11.026 19027050

[B31] Montana MC Cavallone LF Stubbert KK Stefanescu AD Kharasch ED Gereau RW The metabotropic glutamate receptor subtype 5 antagonist fenobam is analgesic and has improved in vivo selectivity compared with the prototypical antagonist 2-methyl-6-(phenylethynyl)-pyridine J Pharmacol Exp Ther 2009 330 834 843 10.1124/jpet.109.154138 19515968 PMC2729799

[B32] Castroman P Ness TJ Vigor of visceromotor responses to urinary bladder distension in rats increases with repeated trials and stimulus intensity Neurosci Lett 2001 306 97 100 10.1016/S0304-3940(01)01886-9 11403967

[B33] Ness TJ Lewis-Sides A Castroman P Characterization of pressor and visceromotor reflex responses to bladder distention in rats: sources of variability and effect of analgesics J Urol 2001 165 968 974 10.1016/S0022-5347(05)66586-X 11176524

[B34] Ness TJ Elhefni H Reliable visceromotor responses are evoked by noxious bladder distention in mice J Urol 2004 171 1704 1708 10.1097/01.ju.0000116430.67100.8f 15017270

[B35] Ness TJ Richter HE Varner RE Fillingim RB A psychophysical study of discomfort produced by repeated filling of the urinary bladder Pain 1998 76 61 69 10.1016/S0304-3959(98)00023-2 9696459

[B36] Birder LA Urothelial signaling Auton Neurosci 2010 153 33 40 10.1016/j.autneu.2009.07.005 19666243 PMC2818048

[B37] Zhu CZ Wilson SG Mikusa JP Wismer CT Gauvin DM Lynch JJ Wade CL Decker MW Honore P Assessing the role of metabotropic glutamate receptor 5 in multiple nociceptive modalities Eur J Pharmacol 2004 506 107 118 10.1016/j.ejphar.2004.11.005 15588730

[B38] Porter RH Jaeschke G Spooren W Ballard TM Buttelmann B Kolczewski S Peters JU Prinssen E Wichmann J Vieira E Fenobam: a clinically validated nonbenzodiazepine anxiolytic is a potent, selective, and noncompetitive mGlu5 receptor antagonist with inverse agonist activity J Pharmacol Exp Ther 2005 315 711 721 10.1124/jpet.105.089839 16040814

[B39] Guarneri L Poggesi E Angelico P Farina P Leonardi A Clarke DE Testa R Effect of selective antagonists of group I metabotropic glutamate receptors on the micturition reflex in rats BJU Int 2008 102 890 898 10.1111/j.1464-410X.2008.07748.x 18489527

[B40] Larson JA Ogagan PD Chen G Shen B Wang J Roppolo JR de Groat WC Tai C Involvement of metabotropic glutamate receptor 5 in pudendal inhibition of nociceptive bladder activity in cats J Physiol 2011 589 5833 5843 22005674 10.1113/jphysiol.2011.215657 PMC3249053

[B41] Hopkins WJ Gendron-Fitzpatrick A Balish E Uehling DT Time course and host responses to Escherichia coli urinary tract infection in genetically distinct mouse strains Infect Immun 1998 66 2798 2802 9596750 10.1128/iai.66.6.2798-2802.1998 PMC108272

[B42] Cervero F Laird JM Visceral pain Lancet 1999 353 2145 2148 10.1016/S0140-6736(99)01306-9 10382712

[B43] Robinson DR Gebhart GF Inside information: the unique features of visceral sensation Mol Interv 2008 8 242 253 10.1124/mi.8.5.9 19015388 PMC2732716

[B44] Gebhart GF Pathobiology of visceral pain: molecular mechanisms and therapeutic implications IV. Visceral afferent contributions to the pathobiology of visceral pain Am J Physiol Gastrointest Liver Physiol 2000 278 834 838 10.1152/ajpgi.2000.278.6.G834 10859211

[B45] Gebhart GF Descending modulation of pain Neurosci Biobehav Rev 2004 27 729 737 10.1016/j.neubiorev.2003.11.008 15019423

[B46] Kolber BJ Montana MC Carrasquillo Y Xu J Heinemann SF Muglia LJ Gereau RW Activation of metabotropic glutamate receptor 5 in the amygdala modulates pain-like behavior J Neurosci 2010 30 8203 8213 10.1523/JNEUROSCI.1216-10.2010 20554871 PMC2898903

[B47] Walker K Reeve A Bowes M Winter J Wotherspoon G Davis A Schmid P Gasparini F Kuhn R Urban L mGlu5 receptors and nociceptive function II. mGlu5 receptors functionally expressed on peripheral sensory neurones mediate inflammatory hyperalgesia Neuropharmacology 2001 40 10 19 10.1016/S0028-3908(00)00114-3 11077066

[B48] Fisher K Lefebvre C Coderre TJ Antinociceptive effects following intrathecal pretreatment with selective metabotropic glutamate receptor compounds in a rat model of neuropathic pain Pharmacol Biochem Behav 2002 73 411 418 10.1016/S0091-3057(02)00832-8 12117596

[B49] Varner AE Beneficial effect of nonsteroidal anti-inflammatory drugs and cyclooxygenase-2 inhibitors in patients with asthma during viral infection J Infect Dis 2002 186 723 724 12195364 10.1086/342392

[B50] Lu YM Jia Z Janus C Henderson JT Gerlai R Wojtowicz JM Roder JC Mice lacking metabotropic glutamate receptor 5 show impaired learning and reduced CA1 long-term potentiation (LTP) but normal CA3 LTP J Neurosci 1997 17 5196 5205 9185557 10.1523/JNEUROSCI.17-13-05196.1997 PMC6573299

[B51] Bradbury MJ Campbell U Giracello D Chapman D King C Tehrani L Cosford ND Anderson J Varney MA Strack AM Metabotropic glutamate receptor mGlu5 is a mediator of appetite and energy balance in rats and mice J Pharmacol Exp Ther 2005 313 395 402 15590770 10.1124/jpet.104.076406

[B52] Evans MS Reid KH Sharp JB Jr Dimethylsulfoxide (DMSO) blocks conduction in peripheral nerve C fibers: a possible mechanism of analgesia Neurosci Lett 1993 150 145 148 10.1016/0304-3940(93)90522-M 8469412

[B53] Hooton TM Stamm WE Diagnosis and treatment of uncomplicated urinary tract infection Infect Dis Clin North Am 1997 11 551 581 10.1016/S0891-5520(05)70373-1 9378923

[B54] Rudick CN Billips BK Pavlov VI Yaggie RE Schaeffer AJ Klumpp DJ Host-pathogen interactions mediating pain of urinary tract infection J Infect Dis 2010 201 1240 1249 10.1086/651275 20225955 PMC2841043

[B55] Neugebauer V Li W Bird GC Bhave G Gereau RW Synaptic plasticity in the amygdala in a model of arthritic pain: differential roles of metabotropic glutamate receptors 1 and 5 J Neurosci 2003 23 52 63 12514201 10.1523/JNEUROSCI.23-01-00052.2003 PMC6742141

[B56] Dolan S Kelly JG Monteiro AM Nolan AM Up-regulation of metabotropic glutamate receptor subtypes 3 and 5 in spinal cord in a clinical model of persistent inflammation and hyperalgesia Pain 2003 106 501 512 10.1016/j.pain.2003.09.017 14659534

[B57] Foxman B Brown P Epidemiology of urinary tract infections: transmission and risk factors, incidence, and costs Infect Dis Clin North Am 2003 17 227 241 10.1016/S0891-5520(03)00005-9 12848468

[B58] Zhang L Foxman B Molecular epidemiology of Escherichia coli mediated urinary tract infections Front Biosci 2003 8 e235 e244 10.2741/1007 12456300

[B59] Ferraguti F Shigemoto R Metabotropic glutamate receptors Cell Tissue Res 2006 326 483 504 10.1007/s00441-006-0266-5 16847639

[B60] Li W Neugebauer V Differential roles of mGluR1 and mGluR5 in brief and prolonged nociceptive processing in central amygdala neurons J Neurophysiol 2004 91 13 24 13679408 10.1152/jn.00485.2003

[B61] Jia H Rustioni A Valtschanoff JG Metabotropic glutamate receptors in superficial laminae of the rat dorsal horn J Comp Neurol 1999 410 627 642 10.1002/(SICI)1096-9861(19990809)410:4<627::AID-CNE9>3.0.CO;2-8 10398053

[B62] Valerio A Rizzonelli P Paterlini M Moretto G Knopfel T Kuhn R Memo M Spano P mGluR5 metabotropic glutamate receptor distribution in rat and human spinal cord: a developmental study Neurosci Res 1997 28 49 57 10.1016/S0168-0102(97)01175-9 9179880

[B63] Karim F Wang CC Gereau RW Metabotropic glutamate receptor subtypes 1 and 5 are activators of extracellular signal-regulated kinase signaling required for inflammatory pain in mice J Neurosci 2001 21 3771 3779 11356865 10.1523/JNEUROSCI.21-11-03771.2001 PMC6762705

[B64] Li Z Ji G Neugebauer V Mitochondrial reactive oxygen species are activated by mGluR5 through IP3 and activate ERK and PKA to increase excitability of amygdala neurons and pain behavior J Neurosci 2011 31 1114 1127 10.1523/JNEUROSCI.5387-10.2011 21248136 PMC3073477

[B65] Zimmermann M Ethical guidelines for investigations of experimental pain in conscious animals Pain 1983 16 109 110 10.1016/0304-3959(83)90201-4 6877845

[B66] Ness TJ Gebhart GF Interactions between visceral and cutaneous nociception in the rat. I. Noxious cutaneous stimuli inhibit visceral nociceptive neurons and reflexes J Neurophysiol 1991 66 20 28 1919667 10.1152/jn.1991.66.1.20

[B67] Hung CS Dodson KW Hultgren SJ A murine model of urinary tract infection Nat Protoc 2009 4 1230 1243 10.1038/nprot.2009.116 19644462 PMC2963178

[B68] Lai HH Boone TB Yang G Smith CP Kiss S Thompson TC Somogyi GT Loss of caveolin-1 expression is associated with disruption of muscarinic cholinergic activities in the urinary bladder Neurochem Int 2004 45 1185 1193 10.1016/j.neuint.2004.06.016 15380628

